# A chemically-defined plastic scaffold for the xeno-free production of human pluripotent stem cells

**DOI:** 10.1038/s41598-022-06356-8

**Published:** 2022-02-15

**Authors:** Eiko Shimizu, Hiroki Iguchi, Minh Nguyen Tuyet Le, Yuta Nakamura, Daigo Kobayashi, Yuhei Arai, Kenta Takakura, Seiko Benno, Noriko Yoshida, Masayoshi Tsukahara, Satoshi Haneda, Kouichi Hasegawa

**Affiliations:** 1 Institute for Integrated Cell-Material Sciences (iCeMS), Institute for Advanced Study, Kyoto University, Yoshida-Ushinomiya-cho, Sakyo-ku, Kyoto, 606-8501 Japan; 2 Sekisui Chemical Co., Ltd., 2-1 Hyakuyama, Shimamoto-cho, Mishima-gun, Osaka, 618-0021 Japan; 3 CiRA Foundation, Kyoto University, 53 Shogoin-Kawara-cho, Sakyo-ku, Kyoto, 606-8397 Japan

**Keywords:** Biotechnology, Stem cells, Materials science

## Abstract

Clinical use of human pluripotent stem cells (hPSCs) is hampered by the technical limitations of their expansion. Here, we developed a chemically synthetic culture substrate for human pluripotent stem cell attachment and maintenance. The substrate comprises a hydrophobic polyvinyl butyral-based polymer (PVB) and a short peptide that enables easy and uniform coating of various types of cell culture ware. The coated ware exhibited thermotolerance, underwater stability and could be stored at room temperature. The substrate supported hPSC expansion in combination with most commercial culture media with an efficiency similar to that of commercial substrates. It supported not only the long-term expansion of examined iPS and ES cell lines with normal karyotypes during their undifferentiated state but also directed differentiation of three germ layers. This substrate resolves major concerns associated with currently used recombinant protein substrates and could be applied in large-scale automated manufacturing; it is suitable for affordable and stable production of clinical-grade hPSCs and hPSC-derived products.

## Introduction

Human pluripotent stem cells (hPSCs), including human embryonic stem cells (hESCs) and induced pluripotent stem cells (hiPSCs), can infinitely self-renew and differentiate into all major lineages of somatic cells in the human body^1–4^. These characteristics make them particularly suitable for applications in regenerative medicine and transplantation therapy. In order to achieve the industrialization of regenerative medicine, there are many problems related to the culture of hPSCs ranging from cost and stability to safety. Moreover, efficient and low-cost culture substrates that facilitate the derivation and large-scale expansion of quality-controlled hPSCs without direct or indirect exposure to xenobiotics are lacking. Although various xeno-free hPSC culture substrates have been developed and are commercially available, most of these require the inclusion of particular recombinant human extracellular matrix (ECM) proteins^5,6^ like vitronectin, laminins, or their fragments^7–9^. These substrates require a laborious and time consuming coating, are thermosensitive and expensive, hindering the cost-effective scale-up of hPSC production for clinical and industrial applications. They are purified from bacteria or animal cells under quality and safety control procedures suitable for clinical cell-based applications. Although several protein-free substrates have been reported^10–16^, these have variable potency and have not yet been commercialized. In addition, during the transition from small laboratory culture to large cell manufacturing, a scale-appropriate cell culture container must be applied. Many scale-appropriate cell culture containers for mass production have already been proposed. However, different cell culture containers have different degrees of surface modification and ECM adsorption^17,18^, so culture performance may not be consistent. Therefore, for smooth transition from the laboratory to the manufacture scale, a uniform culture surface would be highly beneficial. Thus, synthetic substrates that retain their cell culture properties regardless of the culture container may represent a valuable and affordable solution to establish large-scale quality-controlled hPSC production.

**Figure 1:**
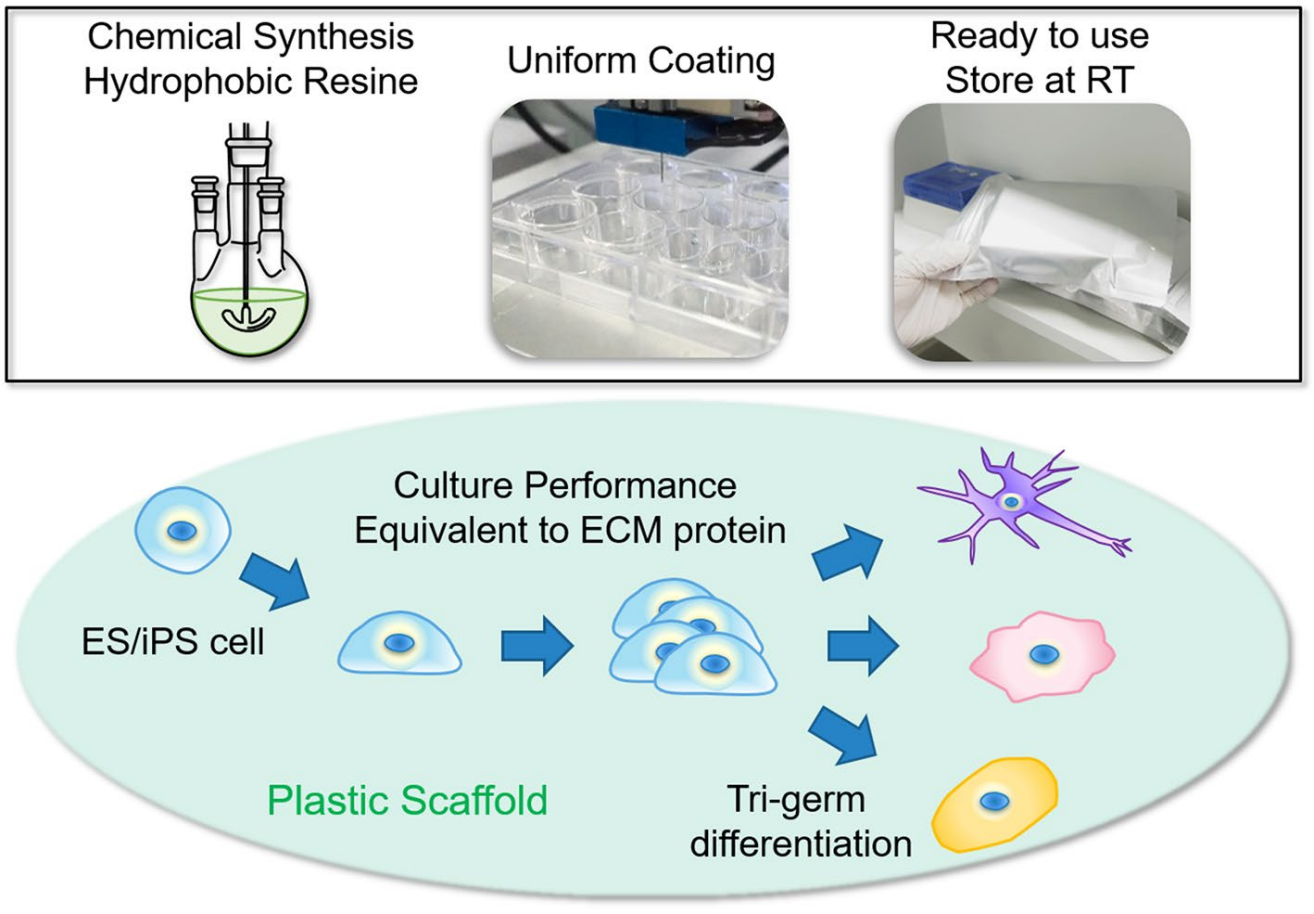
Schematic illustration of the human pluripotent stem cell culture using chemically-defined plastic scaffold.

Herein, we report a novel synthetic chemical culture substrate consisting of a polyvinyl butyral-based polymer (PVB) and a short peptide, named Chemically-defined Peptide-PVB scaffold (CPB scaffold) that can resolve the major problems associated with current recombinant protein substrates (Fig. 1). We have focused on PVB because not only it is a derivative polymer of polyvinyl alcohol, which shows biocompatibility, but it is also presents interfacial adhesiveness between heterogeneous materials. In order to improve cell adhesion, we designed a novel PVB modified with a peptide containing the RGD sequence. Our results show that the modified PVB retained its characteristics on various containers, and showed great properties for long-term cell culture, proving that it might be suitable for large-scale and clinical grade hPSC production.

## Results

### Development of polyvinyl butyral-based polymer substrate suitable for human pluripotent stem cell culture

**Figure 2:**
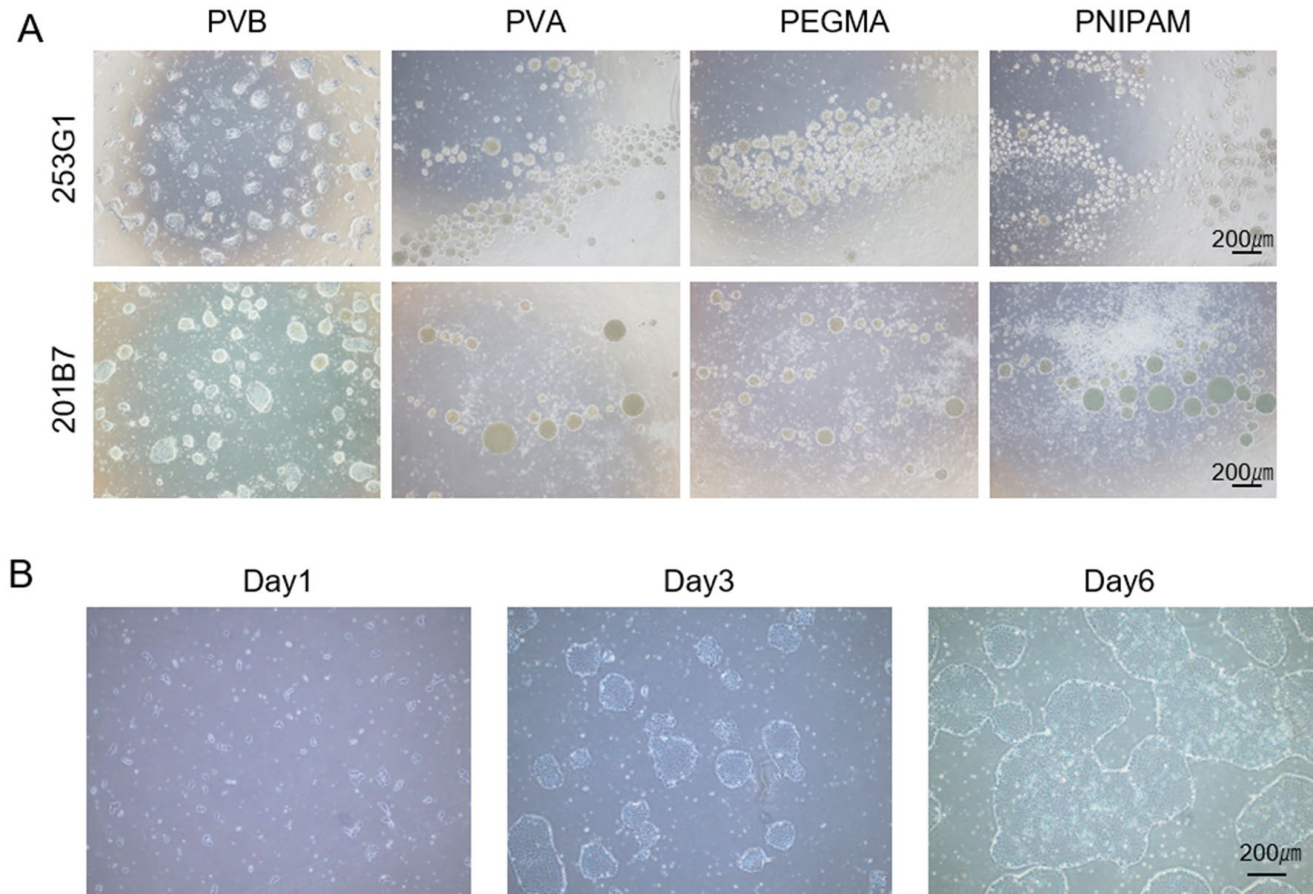
Human induced pluripotent stem cell culture on polyvinyl butyral-based substrate compared with other synthetic chemical substrates. (**A**) 253G1 and 201B7 colony morphology on PVB, PVA, PEGMA and PNIPAM in TeSR-E8 medium. 253G1 cells show the state on day 4, and 201B7 cells show the state on day 7. TeSR-E8 medium was used for both. (**B**) 253G1 colony morphology on CPB scaffold during five days in TeSR-E8 medium. All scale bars are 200 µm.

To develop an affordable and stable culture substrate for hPSCs, we first screened general synthetic polymers suitable for adhesive culture of iPSCs. We started screening PVB polymer substrate to evaluate its surface affinity with iPSCs. Since most of the iPSCs were detached after the medium exchange on day 1, we supplemented the medium with ROCK inhibitor throughout the screening, which is known to support hPSC survival^19^ (Fig. 2A). The difference in affinity between the cells and the resin surface showed that PVB is a better suitable base polymer than others including polyvinyl alcohol (PVA)^20–24^, poly[poly (ethylene glycol) monomethacrylate] (PEGMA)^25^ and poly (N-isopropylacrylamide) (PNIPAM)^26^, which are reported as a polymers applicable for hPSC culture. PVB has in the same main chain a butyl group, which is a hydrophobic group, and a hydroxyl group, which is a hydrophilic group (Figure S1A). However, the contact angle measurement revealed that PVB is more hydrophobic than the other polymers (Table S1). This suggests that iPSCs remain attached through the hydrophobic interaction between the cell basement membrane and PVB. The cells on PVB could be maintained for more than 10 passages (Figure S1B). PVB can be easily dissolved in polar solvents like alcohol, and forms an approximately 100 nm thick uniform film on polystyrene ware just by precast coating of the dissolved solution (details were described in the Experimental Procedures). By combining PVB’s easy and wide usability and our screening results, we concluded that we have found a superior polymer for hPSC culture.

**Figure 3:**
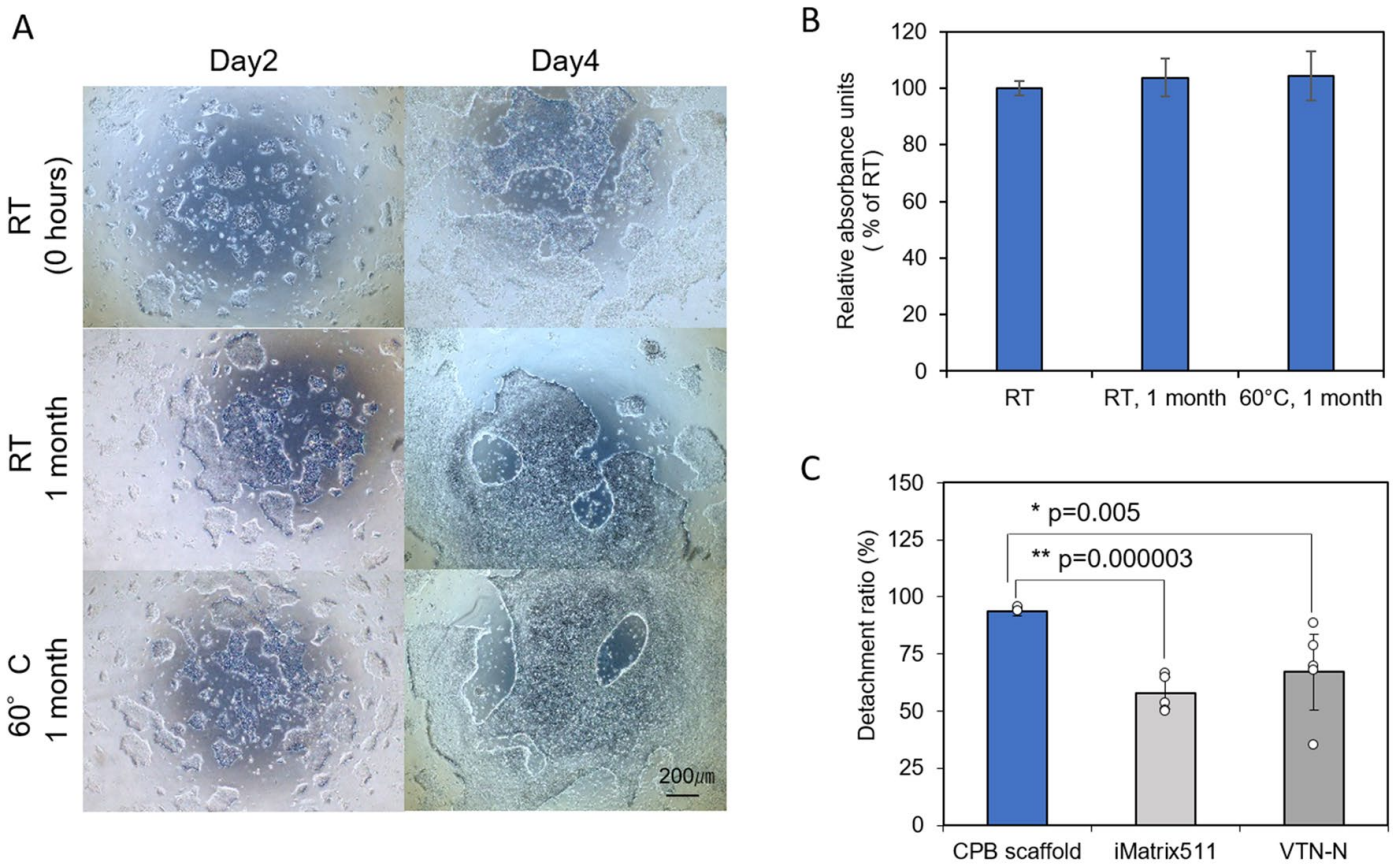
Temperature effect on peptide-polyvinyl butyral-based coated tissue culturing polystyrene and analysis of detachment of cells on different substrates. (**A**) 253G1 colony morphology using incubated CPB scaffold at room temperature and 60 ºC for 1 month, (**B**) Relative absorbance unit of 253G1 after five days of culturing in TeSR-E8 medium after seeding 5 × 10^4^ cells on CPB scaffold. Data are presented as mean ± SD of three independent experiments. (**C**) Detachment ratio of 253G1 cells cultured on CPB scaffold compared with VTN-N and iMatrix 511. Scale bars is 200 µm. Data are presented as mean ± SD of six independent experiments (*P < 0.05 compared to VTN-N, **P < 0.005 compared to iMatrix511).

We found several differences between hPSCs on ECM proteins and on PVB polymer. hPSCs on widely used ECM proteins such as laminin or vitronectin, form a flat monolayer of colonies, and maintain their cell-ECM adhesion independently of the presence of the ROCK inhibitor after passaging procedures. However, the cells on PVB formed multilayer dense colonies (Fig. 2A and S1B), and maintained their attachment depending on the presence of ROCK inhibitor even after passaging. Interestingly, the cells on the PVB polymer detach rather than die after removal of the ROCK inhibitor a few days after passaging. This observation is consistent with ROCK inhibitor’s known functions, not only preventing dissociation-mediated apoptosis in hPSCs^19^, but also promoting both cell–cell and cell-substrate adhesion^27–29^. The ROCK inhibitor dependency suggests that cell-PVB adhesiveness is not sufficient to maintain cell attachment in the absence of ROCK inhibitor. We hypothesized that the cell-PVB adhesiveness force may be too weak to maintain cells as a monolayer and it is overcome by cell–cell adhesion force promoting multilayers. There are many strategies to enhance cell-substrate attachment^30,31^. We tested a synthetic short peptide motif in the fibronectin protein to enhance cell adhesiveness of PVB. As we expected, the cells on this Chemically-defined Peptide-PVB scaffold (CPB scaffold) formed flat colonies, remain attached, and expanded independent of ROCK inhibitor (Fig. 2B). Additionally, the CPB scaffold coating is thermostable; its performance was maintained even after 1 month of storage at 60 °C (Fig. 3A and B). The cells on the CPB scaffold were detached and harvested from the culture plate by calcium and magnesium chelators such as EDTA or sodium citrate solution (Fig. 3C). The harvesting of cells on CPB scaffold was much easier and milder than that on recombinant ECM proteins including vitronectin or laminin. Notably, the CPB scaffold coating is much more uniform than that of large biomaterials, such as laminin or vitronectin, which coat unevenly at a microscale level because of meniscus formed in their coating process^17^. Indeed, the cells on the vitronectin-coated plate remained largely unchanged after a short time and mild detachment procedure, while the cells on the CPB scaffold -coated place were almost all uniformly detached (98%) under the same conditions (Fig. 3C and S2 graph).

### CPB scaffold supports human embryonic stem cell and induced pluripotent stem cell lines in combination with various culture media

**Figure 4:**
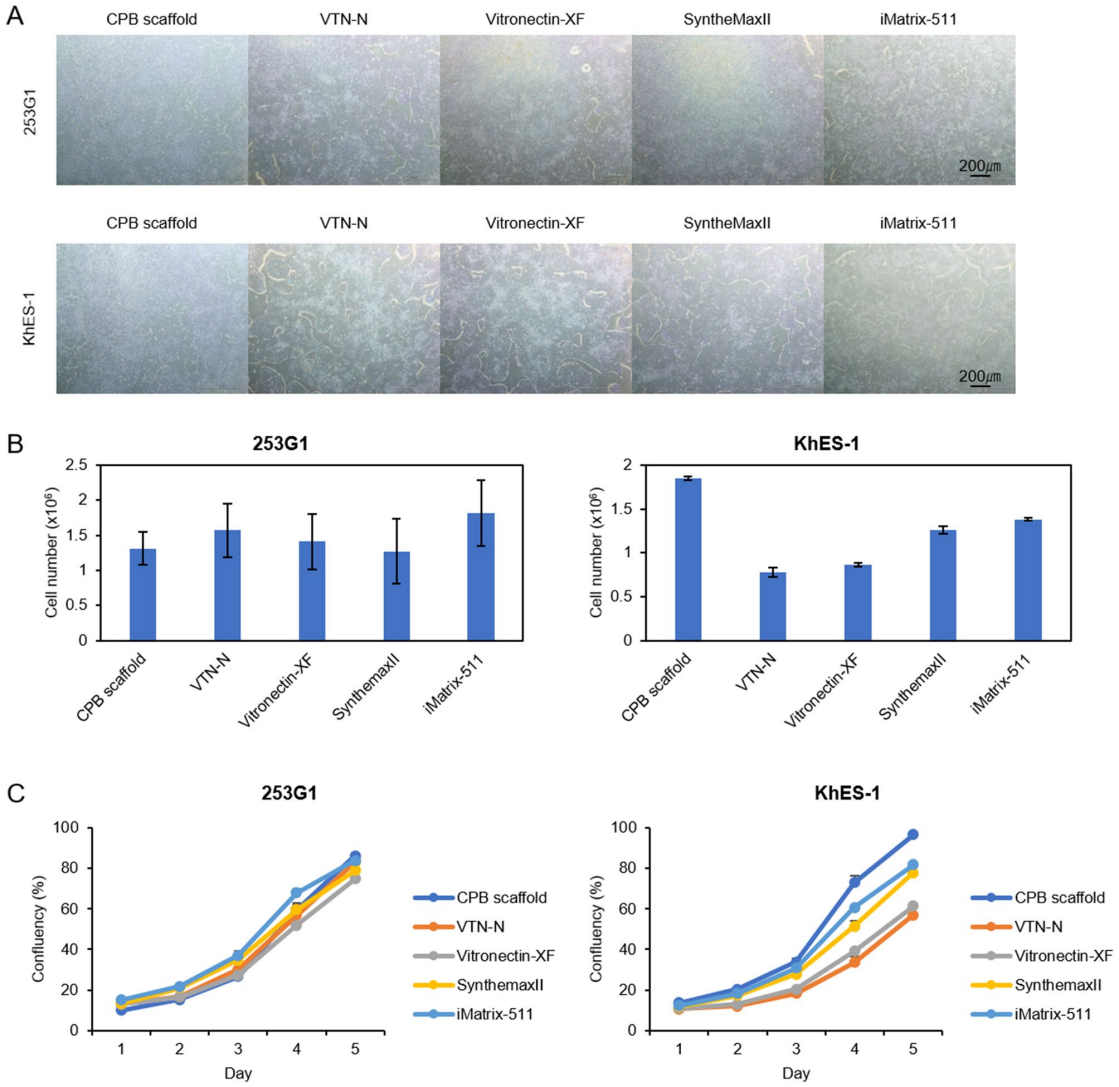
Comparison of CPB scaffold with other commercially available substrates. (**A**) 253G1 and KhES-1 colony morphology on various substrates at 5 days in Essential 8 medium. All scale bars are 200 µm. (**B**) Cell number of 253G1 and KhES-1 at five days of culturing in Essential 8 medium after seeding 5 × 10^4^ cells on various substrate. (**C**) Cell proliferation curves of 253G1 and KhES-1 during 5 days of culturing in Essential 8 medium after seeding 5 × 10^4^ cells on various substrates. Data are presented as mean ± SD of three independent experiments.

To understand the performance and applicable scope of CPB scaffold, we first compared its performance with widely used and commercially available ECM substrates, including a recombinant vitronectin^32^ (Vitronectin-XF; Stem Cell Technologies), a recombinant and truncated vitronectin^32^ (VTN-N, Thermo Fisher Scientific), a synthetic vitronectin peptide-copolymer^33^ (SyntheMax II-SC, Corning), and a recombinant Laminin-511 E8 fragment^8^ (iMatrix-511, Nippi). We used two hiPSC lines and two hESC lines in simple essential 8 culture medium^32^. The cell morphology and colony formation were similar in all cell lines on all substrates, including CPB scaffold (Fig. 4A and S3A). We analyzed the 5 days cellular expansion assay, measured by yield cell number of each culture condition after seeding the same number of cells so that it indicates realistic culture efficiency in combination of cellular attachment, survivals and cell growth effect^34^. This assay showed that CPB scaffold presents similar efficiencies as that of Vitronectin-XF and VTN-N in all examined cell lines (Fig. 4 and S3). The assay also demonstrated that the response to ECM molecules is slightly different among cell lines. CPB scaffold has higher efficiency than SyntheMax II-SC in hESC cell lines, i.e. KhES-1 and H9 (Fig. 4 and S3).

**Figure 5:**
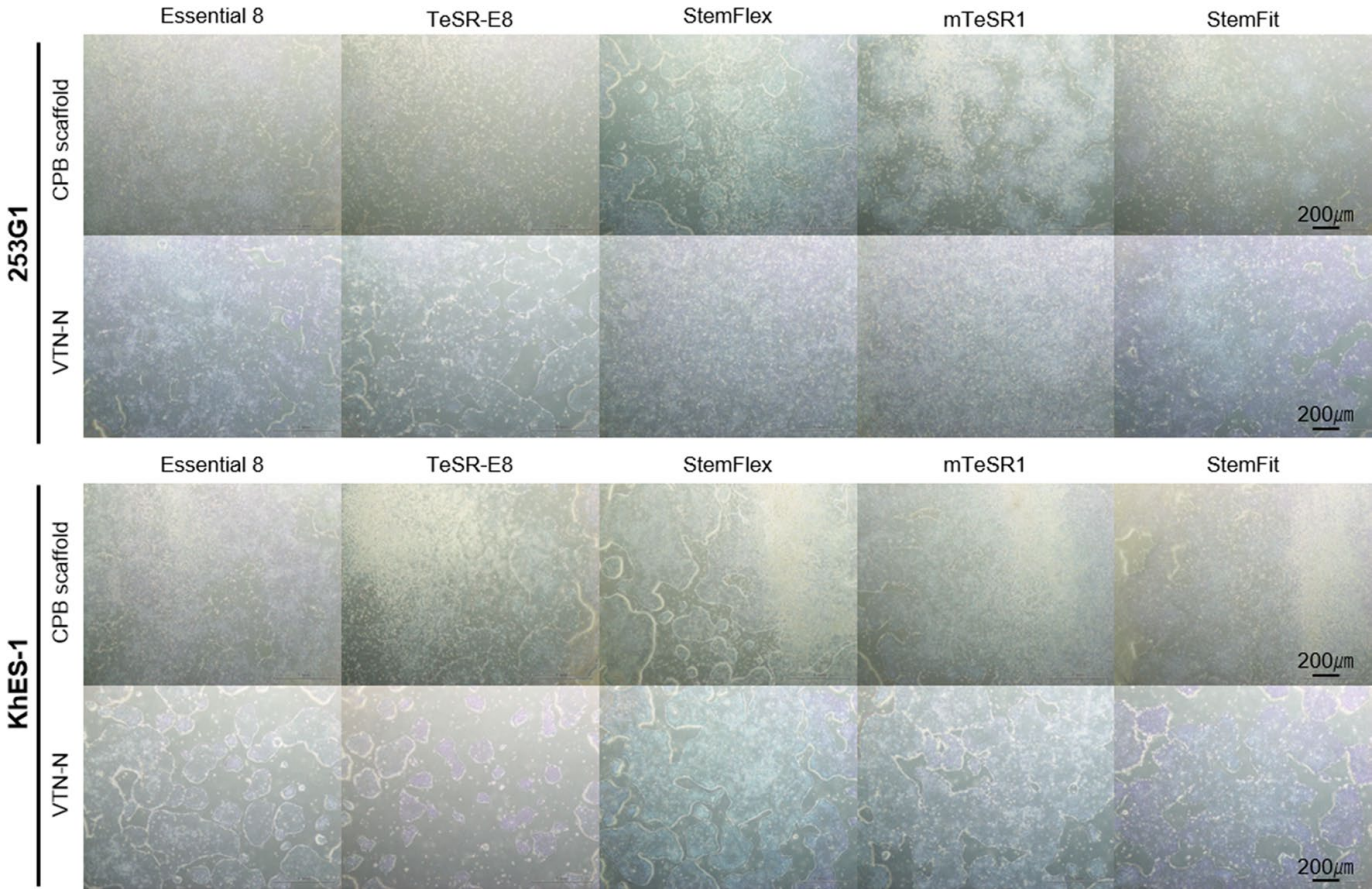
Examination colony formation and cell number on CPB scaffold using multiple media. Colony formation of 253G1 and KhES-1 cells after 5 days in Essential 8, TeSR-E8, StemFlex, mTeSR1, or StemFit cultures. CPB scaffold or VTN-N were used as the culture substrate. All scale bars are 200 µm.

To further examine the versatility of CPB scaffold, it was applied for hPSC culture in a combination of various major and commercially available hPSC media including Essential 8 (Thermo Fisher Scientific), TeSR-E8 (Stem Cell Technologies), StemFlex (Thermo Fisher Scientific), mTeSR1 (Stem Cell Technologies), and StemFit (AK02N, Ajinomoto) medium in comparison with VTN-N. We found that CPB scaffold could support all examined hPSC lines in combination with Essential 8, TeSR-E8, mTeSR1, StemFlex, and StemFit medium at efficiency similar to that of VTN-N (Fig. 5 and S4). CPB scaffold could also support cellular attachment and expansion of H9 and KhES-1 hESC lines. Although there are some differences in the preferences of the multiple hPSC lines, we concluded that CPB scaffold can support hPSC attachment and expansion in a variety of culture media with an efficiency similar to that of commercially available ECM substrates.

### Long-term culture and further characterization of human pluripotent stem cells on the CPB scaffold

**Figure 6:**
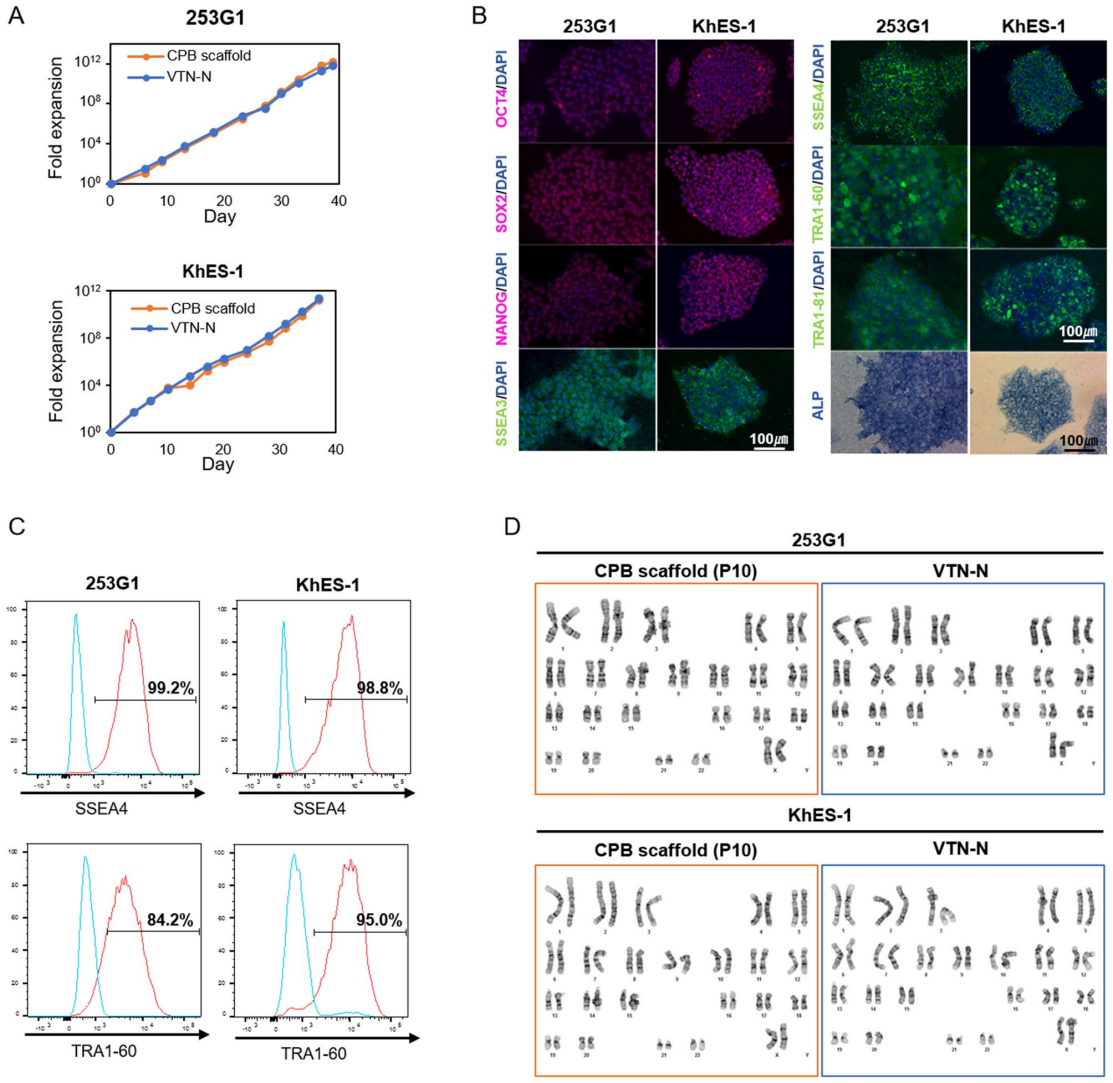
Characterization of human induced pluripotent stem cells and human embryonic stem cells after long-term culture on CPB scaffold. (**A**) Comparison of fold expansion rates of the 253G1 and KhES-1 cell lines on CPB scaffold or VTN-N in Essential 8 medium. Fold expansion is shown in a one-logarithmic graph, and cell passages were performed more than 10 times. The orange circle on the graph shows the number of cells of 253G1 or KhES-1 cultured on CPB scaffold, and the blue circle shows the number of cells cultured on VTN-N. (**B**) Pluripotency marker immunostaining and alkaline phosphatase (ALP) expression analysis. Pluripotency markers are red or green. Nuclear staining is performed with DAPI and shown in blue. ALP is blue in bright field. The scale bar represents 100 µm. (**C**) FACS analysis of pluripotency marker-positive 253G1 and KhES-1 cells. Red and blue histograms show the stained and unstained control populations, respectively. At least 10,000 cells were measured for each sample. The vertical axis shows the mode standardized to 100. The percentage of marker-positive cells is indicated on each graph. (**D**) G-band karyotyping of the cells maintained on CPB scaffold or VTN-N. Using 253G1 cells or KhES-1 cells passaged 10 times or more on CPB scaffold or VTN-N, we observed normal chromosome numbers in 50 cells and performed karyotyping of 20 of them. A typical chromosomal image is shown.

**Figure 7:**
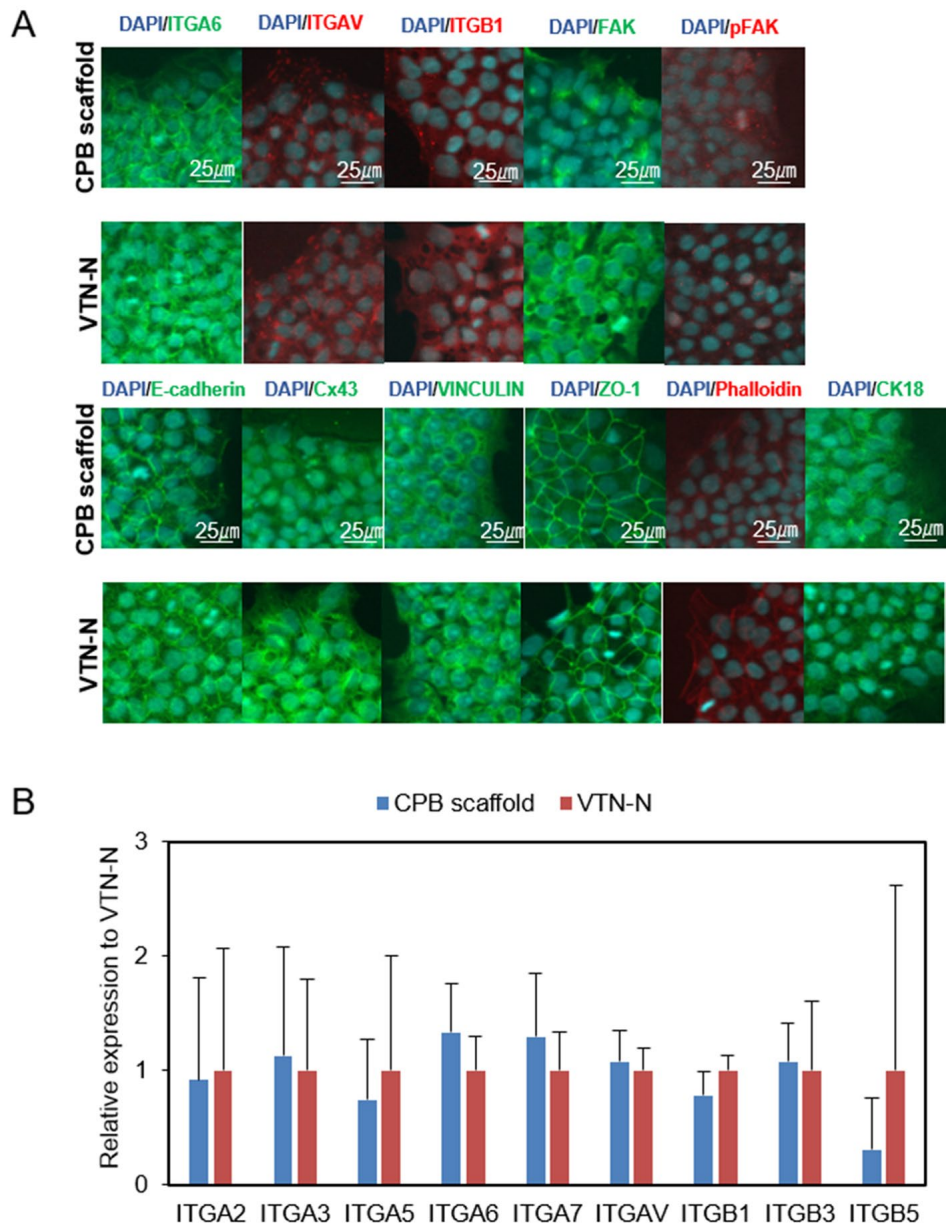
Immunostaining and RT-qPCR of Integrin and other factors. (**A**) Immunofluorescent staining of integrins, focal adhesion molecules, and cytoskeleton proteins of 253G1 cells after more than 10 passages on CPB scaffold or VTN-N. Integrins and other factors are shown in green, red, or orange, and DAPI is shown in blue. Scale bars indicated in the figure. (**B**) Result of RT-qPCR of the relative gene expression of integrins normalized to expression on VTN-N. The vertical axis shows the fold change to VTN-N. Data are presented as mean ± SD of three independent experiments. (*P < 0.05 compared to VTN-N).

To evaluate the sustainability of self-renewal of hPSCs, the CPB scaffold was applied for long-term culture of hiPSCs and hESCs. Growth curves showed that the expansion rate on CPB scaffold was similar to that on VTN-N during the 15 passages (Fig. 6A and S5A). After 10 passages, expression of all the examined pluripotency markers, including OCT4, SOX2, NANOG, SSEA3, SSEA4, TRA-1-60, TRA-1-81, and ALP, remained positive by immunostaining and ALP assay (Fig. 6B and S5B). Flow cytometry analysis revealed that > 95% and > 84% of cells were positive for SSEA4 and TRA-1–60, respectively (Fig. 6C and S5C). These data indicated that hPSCs on CPB scaffold maintained their undifferentiated state. The G-band karyotype demonstrated that hPSCs on CPB scaffold also maintained their karyotype at least after 10 passages (Fig. 6D and S5D). To further investigate the adhesive mechanism of CPB scaffold, gene expression levels and protein expression patterns of adhesive molecules were examined (Fig. 7 and S6). Immunostaining and confocal microscopy analysis revealed that all examined integrin proteins, integrin α6, αV, and β1, which are known to be involved in hPSC adhesion^35–39^ were expressed at similar levels and patterns in cells maintained on CPB scaffold and VTN-N. Focal adhesion, cell–cell adhesion, gap junctions, adherens junctions, tight junctions, microfilament, and intermediate filament were examined by pan- and phosphorylated- Focal adhesion kinase (FAK and p-FAK), E-Cadherin, Connexin 43, Vinculin, ZO-1, F-Actin, and Cyrokeratin 18, respectively. Although the expression pattern of FAK was different between the cells on CPB scaffold and VTN-N, focal adhesion labelled by p-FAK was no significant different between them. There were no significant differences in expression levels and patterns between the cells maintained on CPB scaffold and VTN-N. In addition, there was a slight difference (> 0.5 fold change) in gene expression levels of the major integrin genes in hPSCs maintained on CPB scaffold and VTN-N; including *ITGA2, ITGA3, ITGA5, ITGA6, ITGA7, ITAGV, ITGB1, ITGB2*, and *ITGB5*. These data suggest that hPSCs attach to the CPB scaffold via a similar adhesive mechanism of attachment to vitronectin.

**Figure 8:**
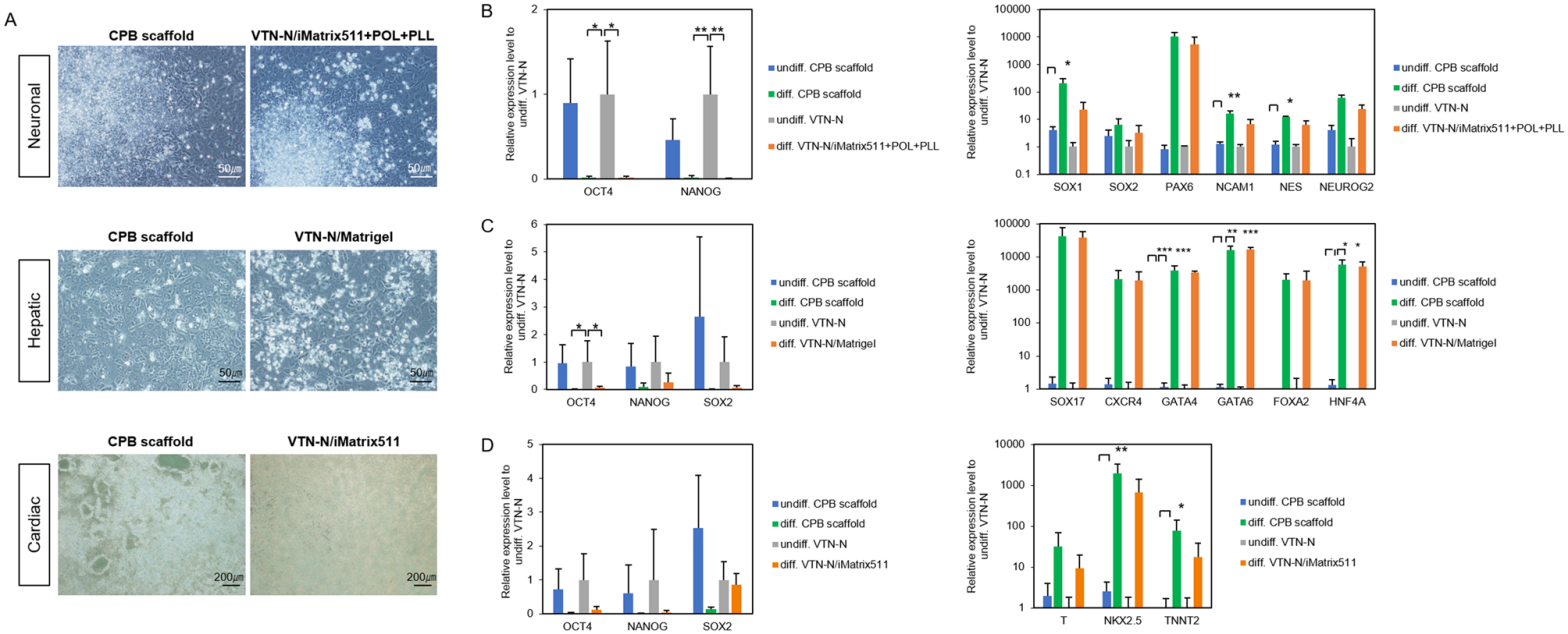
Differentiation of human induced pluripotent stem cells on CPB scaffold into three-germ layers. (**A**) Representative images of differentiated 253G1 cells at 6–7 days after neuronal ectoderm (top), hepatic endoderm (middle), and cardiac mesoderm (bottom) induction. Left images show the differentiated cells on CPB scaffold induced from the cells maintained on CPB scaffold more than 10 passages before differentiation induction. Right images show differentiated cells on control differentiation substrate according to each protocol from the cells maintained on the control maintenance substrate VTN-N. The control differentiation substrate was Laminin/poly-L-ornithine/poly-L-lysin (iMatrix511/POL/PLL) for neural, Matrigel for hepatic, and laminin (iMatrix-511) for cardiac differentiation. Scale bars are indicated in the images. (**B**) Expression level of undifferentiated (OCT4 and NANOG) and neural lineage (SOX1, SOX2, PAX6, NCAM1, NES, and NEUROG2) marker genes in the neuro-ectodermal differentiation. (**C**) Expression level of undifferentiated (OCT4, NANOG, and SOX2) and hepatic lineage (SOX17, CXCR4, GATA4, GATA6m FOXA2, and HNF4A) maker genes in the hepatic endoderm differentiation. (**D**) Expression level of undifferentiated (OCT4, NANOG, and SOX2) and cardiac lineage (T, NKX2.5, and TNNT2) marker genes in the cardiac mesoderm differentiation. All gene expression levels indicated were examined by RT-qPCR, and indicated as relative expression level to undifferentiated 253G1 cells cultured on VTN-N. Data are presented as mean ± SD of three independent experiments. (*P < 0.05, **P < 0.01 and ***P < 0.001 compared to undifferentiated 253G1 cells cultured on VTN-N).

To further assess the CPB scaffold effect on the maintenance of the differentiation potential, the cells were analyzed for downstream directed differentiation toward cell types derived from each three-germ layer after prolonged culture on CPB scaffold in comparison with that on VTN-N (Fig. 8A). Furthermore, we also investigated the applicability of CPB scaffold in the differentiation processes in comparison with conventional substrates suitable for each differentiation, such as laminin/poly-D-lysin/poly-L-ornithine for neuroectoderm^40,41^, Matrigel for foregut endoderm^42^, and laminin for cardiac mesoderm differentiation^43^. To evaluate the applicability of ectoderm differentiation, a standard neural differentiation protocol, dual Smad inhibition^40,44^, was applied after more than 10 passages on CPB scaffold or conventional VTN-N substrate. In the case of cells maintained on the CPB scaffold, neuroectodermal differentiation was performed continuously, while differentiation was performed on laminin/poly-D-lysin/poly-L-ornithine^40,41^ in the case of the cells maintained on VTN-N. On day 7, the cells formed typical neural rosette-like colonies^45^, lost undifferentiated state marker gene expression (*OCT4* and *NANOG*), and started neural lineage marker gene expression (*SOX1, SOX2, PAX6, NCAM1, NES, NEUROG2*) even on CPB scaffold (Fig. 8B). We noted that the expression levels of all the neural lineage marker genes in the cells on CPB scaffold culture were significantly higher than those on conventional matrices. For endoderm differentiation, the cells were subjected to the first part of liver differentiation^42^ on CPB scaffold or conventional ECM substrate (Matrigel). At the late definitive endoderm/early foregut endoderm stage at day 7, the cells acquired angular shapes, expression of undifferentiated state marker genes (*OCT4, NANOG*, and *SOX2*) disappeared, and all examined endodermal marker genes (*SOX17, CXCR4, GATA4, GATA6, FOXA2,* and *HNF4A*) were highly expressed at the same levels in both differentiated cells on CPB scaffold and Matrigel substrates (Fig. 8C). For mesodermal differentiation, a standard cardiac differentiation protocol was applied^43^. At the cardiac mesoderm stage on day 7, the cells differentiated on CPB scaffold almost lost undifferentiated state marker gene expression, whereas the cells differentiated on conventional laminin (iMatrix511) still expressed the undifferentiated and neural marker *SOX2* (Fig. 8D). Both differentiated cells expressed early mesoderm (*T*), cardiac mesoderm (*NKX2.5*), and cardiomyocyte (*TNNT2*) marker gene expression, but the expression levels of these genes were significantly higher in the cells on CPB scaffold than in iMatrix511 cells.

Finally, we applied CPB scaffold to 253G1 hiPSCs for further differentiation of neural progenitor cells, hepatoblasts and cardiomyocytes. In the neural progenitor cell differentiation, more than 90% of the cells on CPB scaffold were positive for beta3 tubulin and formed typical rosette-like neural stem/progenitor colonies at day 12 (Figure S7). Also, in the hepatoblast differentiation, the differentiated cells on CPB scaffold included FOXA2-positive cells at day 10, and population of the FOXA2-positive cells were much higher than that on Matrigel and VTN-N (Figure S8). In the cardiac differentiation at day 14, more than 70% of the cells on CPB scaffold were cTNT-positive and beating (Figure S9 and Movie S1) even though the cTNT-positive cells on iMatrix511 and VTN-N were less than 15%. These results clearly demonstrate that a single chemical CPB scaffold substrate widely and efficiently supports hPSC attachment, growth, and maintenance of pluripotency as well as downstream differentiation into a variety of tissue types.

## Discussion

Although currently available xeno-free defined hPSC culture substrates can be used for clinical purposes, the requirement for high-quality and contamination-free recombinant ECM proteins may pose the biggest challenge when further optimizing affordable and stable culture conditions. We therefore developed a chemical substrate, CPB scaffold, that supports hPSC attachment and self-renewal and can be used as an alternative to recombinant ECM proteins. CPB scaffold represents an improved xeno-free and growth-factor-free defined culture system for the prolonged expansion of hPSCs.

The culture substrate consists of a synthetic PVB polymer, which is optimized for hPSCs culture, and a general synthetic short peptide, which includes five amino acids. This chemical feature enables complete animal-, cell- and xeno-free, quality-controlled production, which can be easily applied for large-scale automated production in factories similar to other plastic ware. Furthermore, this substrate enables uniform and thermotolerant coating of various cell culture vessels, which can be stored at room temperature, similar to normal plastic tissue culture plates. This substrate can be used as a cell adhesive coating by exploiting the characteristics of the hydrophobic resin. CPB scaffold ink that is a solution of CPB scaffold can be applicable to various coating methods such as spray coating, inkjet method, and solvent casting method. This will be enabled stable mass production of cell culture for hPSCs. It can be applied not only in flat containers but also in cell culture carriers having complicated shapes such as films, fibers, hollow fibers, particles, and various devices.

There is a commercially available and synthetic substrate, Synthemax II-SC, which consists of synthetic acrylate polymer and vitronectin-derived long peptides, applicable for hPSC culture. However, Synthemax II-SC needs to be kept at 4 °C and prevented from exsiccation before and after coating. Additionally, the commercial price of Synthemax II-SC is as expensive as other recombinant ECM protein substrates. The main difference between CPB scaffold and Synthemax II-SC is their solubility in water. Synthemax II-SC shows the same solubility in water as other ECM proteins, while our CPB scaffold is insoluble in water and is as easy to handle in polystyrene cell culture containers. Therefore, CPB scaffold is superior to Synthemax II-SC, in thermotolerance and handling. This may be mainly due to the size difference of the peptides used. Taken together, CPB scaffold could overcome most of the disadvantages associated with the production, purification, coating, and storage of recombinant ECM proteins or protein-conjugated polymers as culture substrates.

Our data suggest that hPSCs grown on CPB scaffold are in the same state of pluripotency as those grown on recombinant ECM proteins substrate, at least on VTN-N and in E8 media. The RNA expression level and protein localization of integrins were not significantly different between cells on CPB scaffold and VTN-N substrates. Surprisingly, there were no significant differences in focal adhesion and cytoskeletal molecules. These results suggest that CPB scaffold may maintain cell attachment by a mechanism similar to vitronectin, although CPB scaffold does not include vitronectin, instead, it has a short fibronectin peptide. Interestingly, fibronectin is not suitable for hPSCs culture compared to Vitronectin^46^. Since CPB scaffold is highly flexible and its hydrophobicity and short peptide composition can be easily modified, further mechanistic studies and examinations may lead to the development of more versatile synthetic substrates for hPSCs as well as many other cell types in the future.

We also found that the CPB scaffold matrix had high potential as a substrate for the directed differentiation of hPSCs toward the three germ layers. In general, ECMs used for hPSC maintenance must be switched into specific different substrates in the process of differentiation. Many of these specific substrates for differentiation processes are proteins and still involve problems in practical and clinical applications of hPSCs because they are usually expensive, ununiform, thermosensitive, and contain animal-components. In contrast, in the present study, hPSCs could be differentiated by the continuous use of CPB scaffold. This means that CPB scaffold can be used as a single substrate that covers all processes from hPSC maintenance to differentiation. More importantly, gene expression analysis indicated that the differentiation on CPB scaffold was more efficient compared with that on widely used substrates such as Matrigel and synthetic polymer/ECM protein composites, particularly in the neural and cardiac directions. This might be due to the combined chemical properties of the synthetic polymer and peptide moiety in CPB scaffold. The hydrophobicity of CPB scaffold could also affect the adhesiveness and/or maturation of differentiated cells. Although the exact mechanism that leads to the highly efficient differentiation needs to be further studied, our findings suggest that the CPB scaffold substrate is a suitable and affordable synthetic substrate for the manufacture of hPSCs and hPSC-derived products.

In conclusion, we developed a novel and practical synthetic scaffold, CPB scaffold, for the mass production of hPSCs. This scaffold certainly accelerates the scalable production of hPSCs and will promote the clinical application of hPSC-derived functional cells.

## Methods

### Human pluripotent stem cell lines

The hESC lines H9 (WiCell, Madison, WI, USA) and KhES-1 (RIKEN, Tsukuba, Japan), and hiPSC lines 253G1 (RIKEN) and 201B7 (RIKEN) were maintained using the standard enzymatic bulk expansion method. All cells were maintained in E8 medium (TeSR-E8, Stem Cell Technologies or Essential-8, Thermo Fisher Scientific) with laminin 511-E8 (iMatrix-E8, Nippi, Japan) substrate and hypertonic sodium citrate passage solution as previously described^34^. All cells were confirmed to be negative for mycoplasma contamination every three months with the MycoAlert PLUS Mycoplasma Detection Kit (Lonza, Basel, Switzerland). All experiments using human cells were conducted under the approval of the Institutional Ethics Committee, Kyoto University, Japan. The hESC lines were used in accordance with the Guidelines for the Derivation and Utilization of Human Embryonic Stem Cells of the Ministry of Education, Culture, Sports, Science and Technology, Japan.

### Synthesis of CPB scaffold

Polyvinyl alcohol (PVA), Poly[poly (ethylene glycol) methacrylate] (PEGMA) and Polyvinyl butyral (PVB) was provided by Sekisui Chemical Co., Ltd. (Tokyo, Japan). Poly (N-isopropylacrylamide) (PNIPAM) were purchased from Sigma Aldrich (St. Louis, MO). Polyvinyl butyral was synthesized using a slight modification of previously reported procedures^47^. Acrylic acid-grafted polyvinyl butyral was synthesized by general radical polymerization with polyvinyl butyral and acrylic acid using t-butyl peroxy-2-ethylhexanoate in butanol. Acrylic acid-grafted PVB was cast-coated by a micropipette on a standard tissue culturing polystyrene (TCPS, Corning® Costar® TC-Treated Multiple Well Plates (12-well), Corning Incorporated, New York, USA). Then, peptide-grafted polyvinyl butyral was synthesized by a condensation reaction with acrylic acid-grafted PVB and general RGD motif peptide using 1-Ethyl-3-(3-dimethylaminopropyl)carbodiimide and 1-Hydroxy-7-azabenzotriazole in water at 40 °C overnight. The pre-coated TCPS was vacuum-dried at 60 °C overnight.

### Preparation of polyvinyl butyral-based polymer coated cell culturing plate

Typically, Acrylic acid-grafted PVB powder was dissolved in butanol at 60 °C for 10 h. The PVB concentrations were 1.0 wt%. PVB in butanol was cast-coated by a micropipette on a standard TCPS and dried in a vacuum overnight at 60 °C, then sterilized using a UV irradiator. After RGD motif peptide was condensed to PVB-coated surface, hPSCs were seeded onto these dishes. As conventional polymers, PVA, PNIPAM, and PEGMA were dissolved in methanol, butanol and/or water, and were coated by the same procedure.

### Characterization of polymer coated surface

The hydrophilicity of the polymer surface was analyzed by the water contact angle using the sessile drop method (DMo-901, Kyowa Interface Science Co., Ltd., Saitama, Japan).

### Heat-resistant analysis of CPB scaffold coated tissue culturing polystyrene

CPB scaffold-coated TCPS were incubated in an oven at 25 °C and 60 °C for 1 month. After sterilization by a UV irradiator, these dishes were used for hiPSC culture. As a positive control, CPB scaffold-coated TCPS were directly used for cell culture after the preparation process. After culturing for 5 days, cell proliferation was evaluated using a Cell Counting Kit (CCK-8 kit, Dojindo Laboratories Inc., Kumamoto, Japan) and a microplate reader.

### Detachment of human induced pluripotent stem cells

hiPSC colonies on PVB-based polymer-coated TCPS were washed with PBS and immersed in EDTA solution (0.5 mM, in PBS) for 5 min at 25 °C. After the EDTA solution was removed using medium flush (1 mL), pipetting was performed for five cycles. The obtained cells were counted as *Cell Count 1* by Image Cytometer (NC3000, Chemometec A/S, Allerod, Denmark). Then, PVB-based polymer-coated TCPS was re-immersed in fresh EDTA solution (0.5 mM, in PBS) for 5 min at 37 °C in order to salvage the remaining cells. These salvaged cells were counted as *Cell Count 2*.

Detachment ratio defined as

$$\mathrm{Detachment\,ratio }\,(\mathrm{\%})=\frac{Cell\,count 1}{Cell\,count 1+Cell\,count 2}\times 100$$

### Cell culture on peptide-polyvinyl butyral-based polymer

For the evaluation of CPB scaffold in comparison with other culture substrates, the dissociated cells were suspended in Essential-8 medium and seeded on 12-well plates coated with Vitronectin-N terminal fragment (VTN-N, Life Technologies), vitronectin (Vitronectin XF, Stem Cell Technologies), Synthemax (Synthemax II-SC Substrate, Corning, Corning, NY, USA), Laminin-511 E8 fragment (Laminn-E8, iMatrix, Nippi, Tokyo, Japan), and Laminin-511 (Laminin-5, ReproCell, Kanagawa, Japan). After 5 days of culture, cells were dissociated with 0.5 mM EDTA/PBS and the cell number was counted. For the evaluation of CPB scaffold with various culture media, the cells were dissociated with hypertonic sodium citrate solution as previously described, suspended with Essential-8 (Thermo Fisher Scientific), StemFit AK02 (Ajinomoto, Japan), StemFlex (Thermo Fisher Scientific) or mTeSR1 (Stem Cell Technologies) medium supplemented with ROCK inhibitor (10 μM, Y-27632), and seeded on CPB scaffold coated 12-well plates at a concentration of 1.25 × 10^4^ cells/well. The ROCK inhibitor was removed 24 h after seeding, and the medium was changed daily.

For long-term culture, the cells were maintained in Essential-8 medium on CPB scaffold-coated 12-well plates. VTN-N-coated plates were used as a control/reference. The medium was changed daily, and the cells were passaged with hypertonic sodium citrate solution every 3–4 days.

### Characterization of human pluripotent stem cells maintained on CPB scaffold^34^

Cell confluency (%) was calculated using IncuCyte® software by phase contrast images. Phase contrast imaging was performed using the IncuCyte® S3 Live-Cell Analysis System (Essen Bioscience, Ann Arbor, MI, USA). Cells were scanned approximately every 24 h from 1 to 5 days post seeding.

To assess pluripotency, hPSCs were fixed with 4% paraformaldehyde for 10 min, and ALP activity was determined with a Vector Blue Alkaline Phosphatase Substrate Kit. For immunostaining, the fixed cells were permeabilized with 0.2% Triton X-100 in PBS, and then incubated with primary antibodies against OCT4 (clone C10, Santa Cruz), SOX2 (Y17, Santa Cruz), NANOG (D73G4, Cell Signaling Technology), SSEA-3 (clone 631, Santa Cruz), SSEA-4 (clone MC813, Santa Cruz), TRA-1-60 (sc-21705, Santa Cruz), and TRA-1-81 (sc-21706, Santa Cruz), followed by Dylight 488-conjugated (Jackson Labs, Bar Harbor, ME, USA), Alexa Fluor 488-conjugated (Life Technologies), or 594-conjugated (Life Technologies) secondary antibodies. Marker expression was visualized by fluorescence microscopy (BZ-X800, Keyence, Osaka, Japan).

To examine the marker-positive population, the cells were dissociated with 0.05% TrypLE (Life Technologies), fixed with 2% paraformaldehyde, and immunostained with SSEA-4 and TRA-1-60 followed by Alexa Fluor 488-conjugated antibodies (Life Technologies). The cells were then analyzed by flow cytometry (FACS Canto II, BD Biosciences, San Jose, CA, USA). To normalize and overlay the charts, the number of cells in each bin (the numerical ranges for the parameter on the fluorescence intensity in the X-axis) were divided by the number of cells in the bin that contained the largest number of cells in FlowJo software (TreeStar, Ashland, OR, USA).

For karyotype analysis, hPSCs were incubated in colcemid (KaryoMAX, Life Technologies), dissociated with trypsin/EDTA, treated with a hypotonic solution, and then fixed with Carnoy’s fixative solution. The G-banding karyotype of 50 randomly selected mitotic metaphase nuclei was analyzed by Nihon Gene Research Laboratories (Sendai, Japan).

### Cell adherent and cytoskeletal molecule analysis

For real time quantitative PCR (RT-qPCR) of integrins, the cells were cultured in 12-well plates that were pre-coated with VTN-N or CPB scaffold for 3–4 days and then harvested for RNA extraction using RNeasy Mini Kit (QIAGEN). RNA was then reverse transcribed into cDNA using the SuperScript™ III First-Strand Synthesis System (18080051, Life Technologies). The primers for RT-qPCR is listed in Table S2. PCR data were analyzed using the AB7900 system and RQ Manager software (Applied Biosystems). Marker expression was normalized to cyclophilin A expression.

To detect cell adhesion and cytoskeletal molecules, cells were cultured in an 8-well chamber (SCS-N08, Matsunami) that was pre-coated with VTN-N or CPB scaffold for 3–4 days, then fixed for 15 min at room temperature in 4% paraformaldehyde in PBS. After fixation, cells were permeabilized with 0.2% Triton X-100 in PBS for 5 min. Cells were then rinsed three times with PBS and immersed in blocking buffer for 30 min. The blocking buffer consisted of 2% BSA and 3% of the animal serum from which the secondary antibody was derived. The primary antibody was diluted in blocking buffer and incubated at 4 °C overnight. Cells were rinsed three times before binding with the secondary antibody for 30 min at room temperature. Finally, the cells were rinsed four times with PBS and mounted with Prolong Gold anti-fade reagent with DAPI (P36935, Life Technologies). Cells were observed under a confocal microscope (FV10i).

The first antibodies used for studying the adherent and cytoskeletal molecules include: ITGA6 (sc-19622, Santa Cruz), ITGAV (sc-9969, Santa Cruz), ITGB1 (100562-T46, Sino Bio), FAK (sc-557, Santa Cruz), p-FAK (sc-11765-R, Santa Cruz), E-cadherin (sc-21791, Santa Cruz), Cx43 (14-4759-80, eBioscience), VINCULIN (sc-25336, Santa Cruz), ZO-1 (33–9100, Thermo Fisher Scientific), CK18 (sc-6259, Santa Cruz). The secondary antibodies included Alexa Fluor 488-conjugated (Life Technologies) or 594-conjugated (Life Technologies).

Particularly for phalloidin staining, cells were permeabilized for 5 min, then stained directly with phalloidin conjugated with iFluor 594 (20553, CAY) in one step.

### Differentiation of human induced pluripotent stem cells into neuronal cells, hepatic cells, and cardiomyocytes on CPB scaffold

For targeted differentiation, the cells were cultured following the dual SMAD inhibition neural differentiation protocol^40,44^, sequential hepatic differentiation protocol^42^, and chemically defined cardiac differentiation protocol^43^. The details are as follows: for the control culture of neuronal differentiation, we prepared 12-well plates that were incubated with Poly-D-Lysine (1 μg/cm^2^) and Poly-D-Ornithine (1 μg/cm^2^) for 30 min at room temperature, and washed three times with PBS + (containing Ca and Mg). After that, 12-well plates were coated with iMatrix511 (1 μg/cm^2^). Culture medium of 253G1 cells cultured on CPB scaffold or VTN-N was replaced with PBS + . Cell colonies were dissected into 0.3–0.5 mm square pieces using a 27 gauge needle, and then gently transferred onto CPB scaffold or iMatrix511-coated 12-well plates containing Neural Induction Medium (NIM), supplemented with SB431542 (10 μM) and LDN193189 (100 nM). The NIM contained equal amounts of Neurobasal medium and DMEM/F12, supplemented with 0.3% glucose, 2 mM L-glutamine, 1 × N-2, 0.5 × B27, 1 × ITS-A, and 0.5 × penicillin/streptomycin. The NIM was changed every other day, and the cells were cultured for 7 days. After day 8, the NIM supplemented with FGF2 (20 ng/ml) was changed every other day, and the cells were cultured for 12 days.

For the control culture of hepatic differentiation, the 12-well plates were coated with standard (growth factor-reduced) Matrigel (1:50). 253G1 cells were dissociated into single cells with 1 mM EDTA, and 3.3 × 10^4^ cells per well were cultured in TeSR-E8 medium on 12-well plates. The next day, the cells were cultured in an incubator at 4% O_2_, 5% CO_2_, and 37 °C. After 48 h, the medium was replaced with RPMI1640 containing 1 × GlutaMAX, B27 without insulin, 1% NEAA, 1% penicillin/streptomycin, 100 ng/ml activin A, 50 ng/ml BMP4, 3 μM CHIR99021, and cells were cultured in an incubator at 20% O_2_, 5% CO_2_, and 37 °C. After 24 h, the medium was replaced with RPMI1640 containing GlutaMAX, B27 without insulin, 1% NEAA, 1% penicillin/streptomycin, activin A, and BMP4. Thereafter, the medium was changed daily. From day 8 to day 10, the cells were cultured in an incubator at 5% O_2_, 5% CO_2_, and 37 °C. The DE medium (RPMI1640 containing GlutaMAX, B27 without insulin, 1% NEAA, 1% penicillin/Streptomycin, activin A, and BMP4) supplemented with FGF2 (10 ng/ml) and HGF (10 ng/ml) was changed daily.

For cardiac differentiation, 253G1 cells (4 × 10^5^ cells per well) were seeded in TeSR-E8 medium containing 5 μM Y27632 on 12-well plates coated with CPB scaffold or iMatrix511. The medium was changed daily. Four days later, the medium was changed to 2 mL per well of RPMI1640 containing B27 without insulin and 12 μM CHIR99021 to initiate differentiation. After exactly 24 h, the medium was replaced with 2 mL of RPMI1640 containing B27 without insulin per well. After 48 h (72 h after addition of CHIR99021), 2 μM XAV939 was added to 2 mL RPMI1640/B27 without insulin. Forty-eight hours later, the medium was replaced with RPMI1640/B27 without insulin and cultured until day 7 of differentiation. On day 7 of differentiation, medium was changed to RPMI1640/B27 with insulin every other day.

At 7 days of differentiation, differentiated neuronal, hepatic, or cardiac cells were analyzed by RT-qPCR. For RT-qPCR, total RNA was isolated from hPSCs and reverse transcribed as previously described. RT-qPCR was performed with the following gene-specific primer/probe mixes: OCT4 (POU5F1, Hs01895061_u1), SOX2 (Hs01053049_s1), NANOG (Hs02387400_g1), PAX6 (cat#Hs01088112_m1), NCAM1 (Hs00941821_m1), NES (Hs00707120_s1), NEUROG2 (Hs00702774_s1), CXCR4 (Hs00607978_s1), GATA4 (Hs00171403_m1), GATA6 (Hs00232018_m1), FOXA2 (Hs00232764_m1), HNF4A (Hs00230853_m1), T (Hs00610080_m1), and Cyclophilin A (PP1A, Hs99999904_m1) (TaqMan Gene Expression Assays, Applied Biosystems, Foster City, CA, USA), TaqMan 2 × Master Mix, and ABI Prism 7900 Sequence Detection System (Applied Biosystems) according to the manufacturer’s protocol. To detect NKX2.5 and TNNT2 expression, RT-qPCR amplifications were carried out in 10 μl reactions using the SYBR Green PCR Master Mix (Thermo Fisher Scientific) according to the manufacturer’s instructions. The primer for RT-qPCR^48^ is listed in Table S3. PCR data were analyzed by the Δ/ΔCT method and normalized to cyclophilin A expression with RQ Manager software (Applied Biosystems).

The differentiated 253G1 cells on day 14 (cardiomyocytes), day 10 (hepatoblasts), day 12 (neural cells) were dissociated with TrypLE express (Life Technologies) and then fixed with 2% paraformaldehyde for 2 min at room temperature. The fixed cells were permeabilized with 0.1% Triton-X in 0.5% BSA/PBS at room temperature for 5 min. The cells were stained with the antibodies against TroponinT-C (cTnT, CT3, sc-20025, Santa Cruz) for cardiomyocytes, FOXA2 (EPR4466, ab108422, ABCAM LIMITED) for hepatoblasts, β-III tubulin (Tuj1, MAB1195, R&D Systems) for neural cells. Alexa Fluor 488-conjugated anti-mouse IgG2A secondary antibody (A21131, Life Technologies), Alexa Fluor 488-conjugated anti-rabbit IgG secondary antibody (A21206, Life Technologies) and Alexa Fluor 647-conjugated anti-mouse IgG secondary antibody (A32787, Life Technologies) was used for cTNT, FOXA2 and β-III tubulin, respectively. The population was analyzed by flow cytometry (FACS Canto II, BD Biosciences, or SA3800, SONY). To normalize and overlay the chart, the number of cells in a bin (the numerical ranges for the parameter on the fluorescence intensity in the X-axis) were divided by the number of cells in the bin that contained the largest number of cells in FlowJo software (TreeStar).

### Statistical analysis

Experiments were repeated at least three times. Statistical significance was determined by Student’ s t test for pairwise comparison or one-way analysis of variance followed by Dunnett’s test using JMP pro version 15 (SAS Institute, Inc., Cary, NC, USA) for multiple comparisons. P-values of less than 0.05 were considered statistically significant.

## Supplementary Information

Supplementary Video 1.

Supplementary Information 1.
