# Supplementary material for: A chemically-defined plastic scaffold for the xeno-free production of human pluripotent stem cells
Source: Sci Rep. 2022 Feb 15;12:2516. doi: 10.1038/s41598-022-06356-8 (PMC8847402; doi:10.1038/s41598-022-06356-8)
Supplement: Supplementary file 2 — Supplementary Information 1. [file 41598_2022_6356_MOESM2_ESM.docx]

**Supplementary information**

**
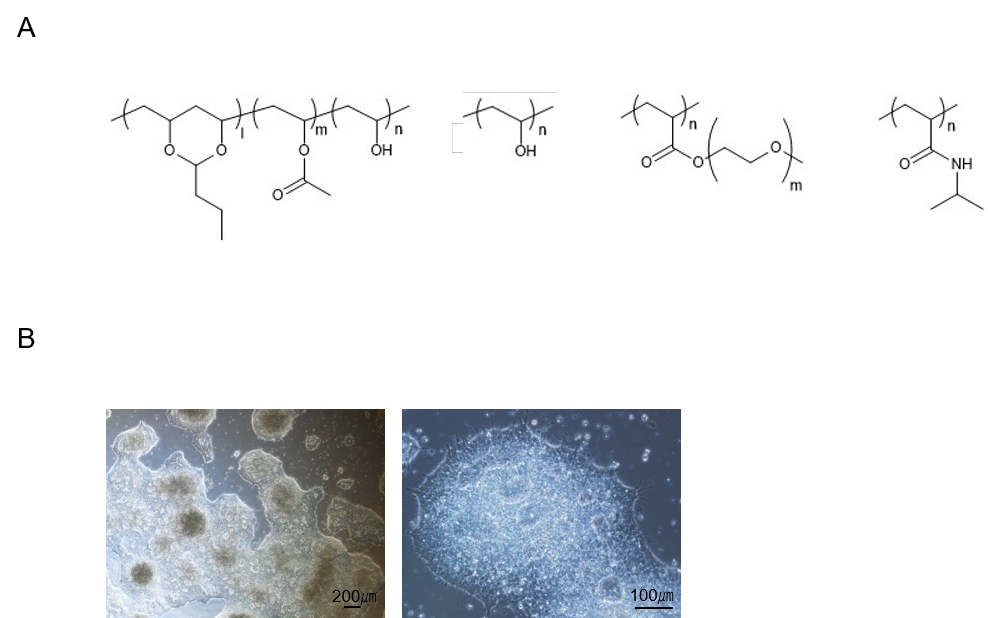
**

**Figure S1.** Properties of polyvinyl butyral-based substrate and other synthetic chemical substrates. Related to **Figure 2**. (A) Chemical structure of PVB, PVA, PEGMA and PNIPAM substrate. (B) The state of 253G1 cells after culturing on PVB for 5 days in TeSR-E8 medium after 10 passaging with ROCK inhibitor is shown.

**
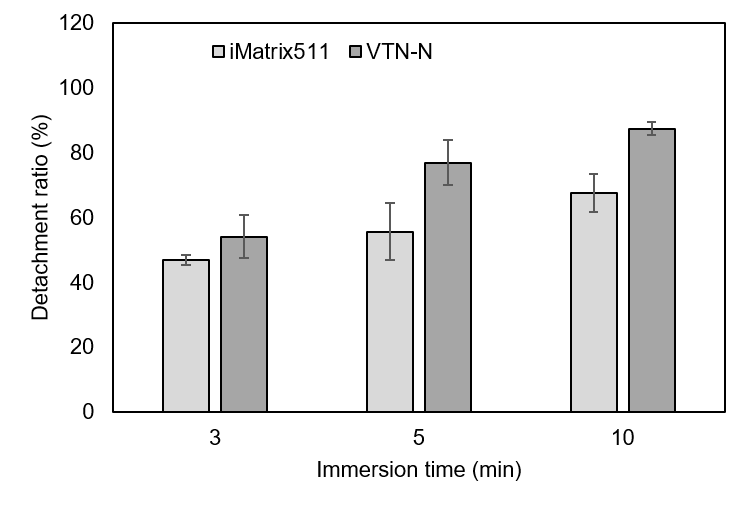
**

**Figure S2.** Relationship between immersion time in EDTA solution and detachment ratio of 253G1 cells cultured on VTN-N or iMatrix511 in TeSR-E8 medium. Related to **Figure 3**. Date are presented as mean ± SD of three independent experiments. (**P*<0.05, and ****P*<0.001 compared to CPB scaffold.)

**
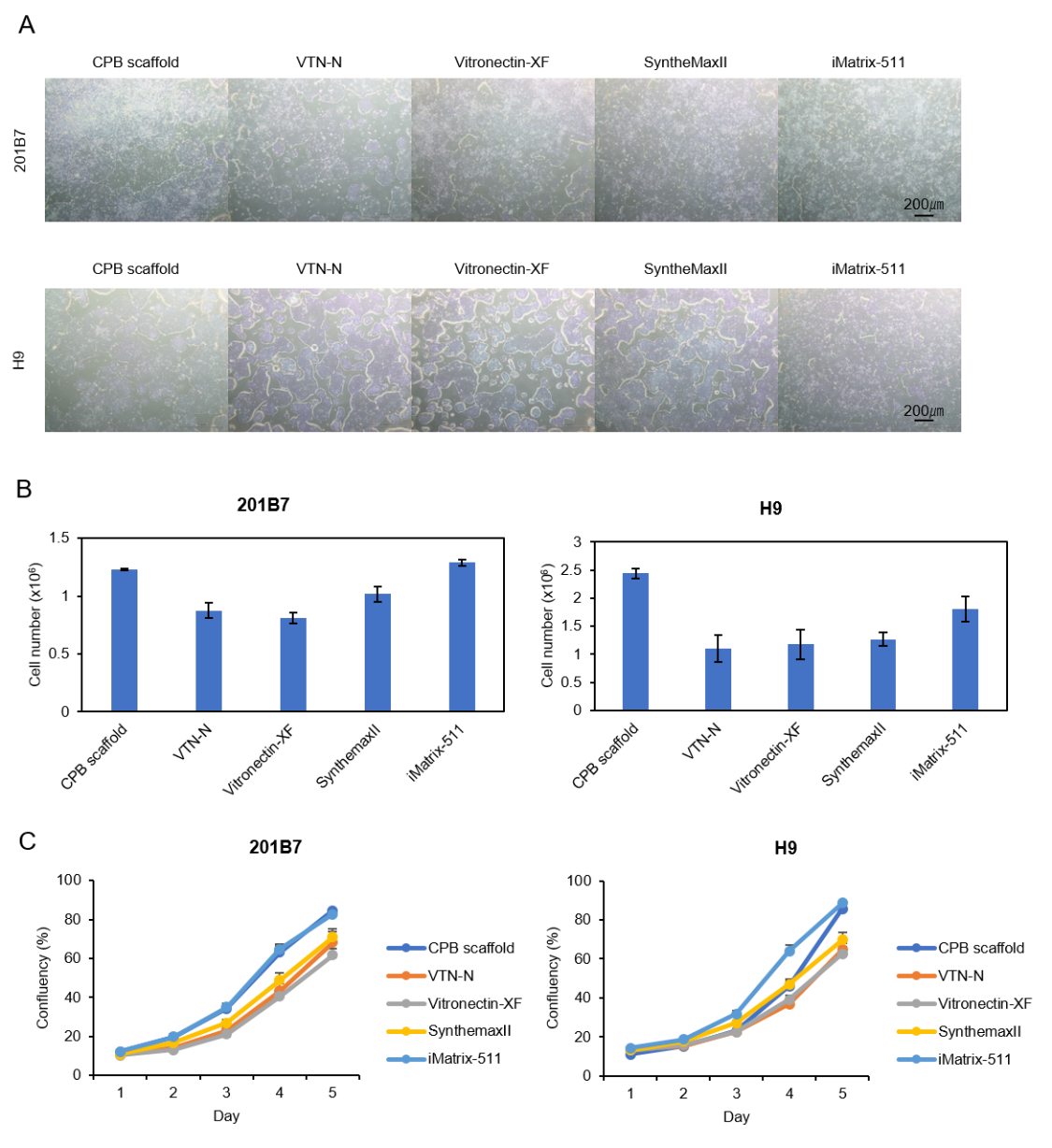
**

**Figure S3.** Comparison of CPB scaffold with other commercially available substrates. Related to **Figure 4**. (A) 201B7 and H9 colony morphology on various substrate at 5 days in Essential 8 medium. All scale bars are 200 µm. (B) Cell number of 253G1 and KhES-1 at five days of culturing in Essential 8 medium after seeding 5 x 10^4^ cells on various substrate. (C) Cell proliferation curves of 201B7 and H9 during five days of culturing in Essential 8 medium after seeding 5 x 10^4^ cells on various substrates. Data are presented as mean ± SD of three independent experiments.

**
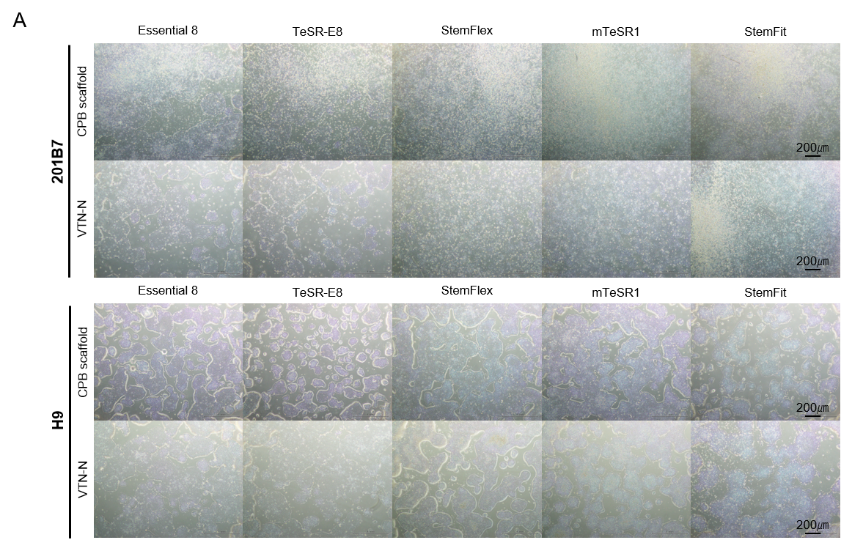
**

**
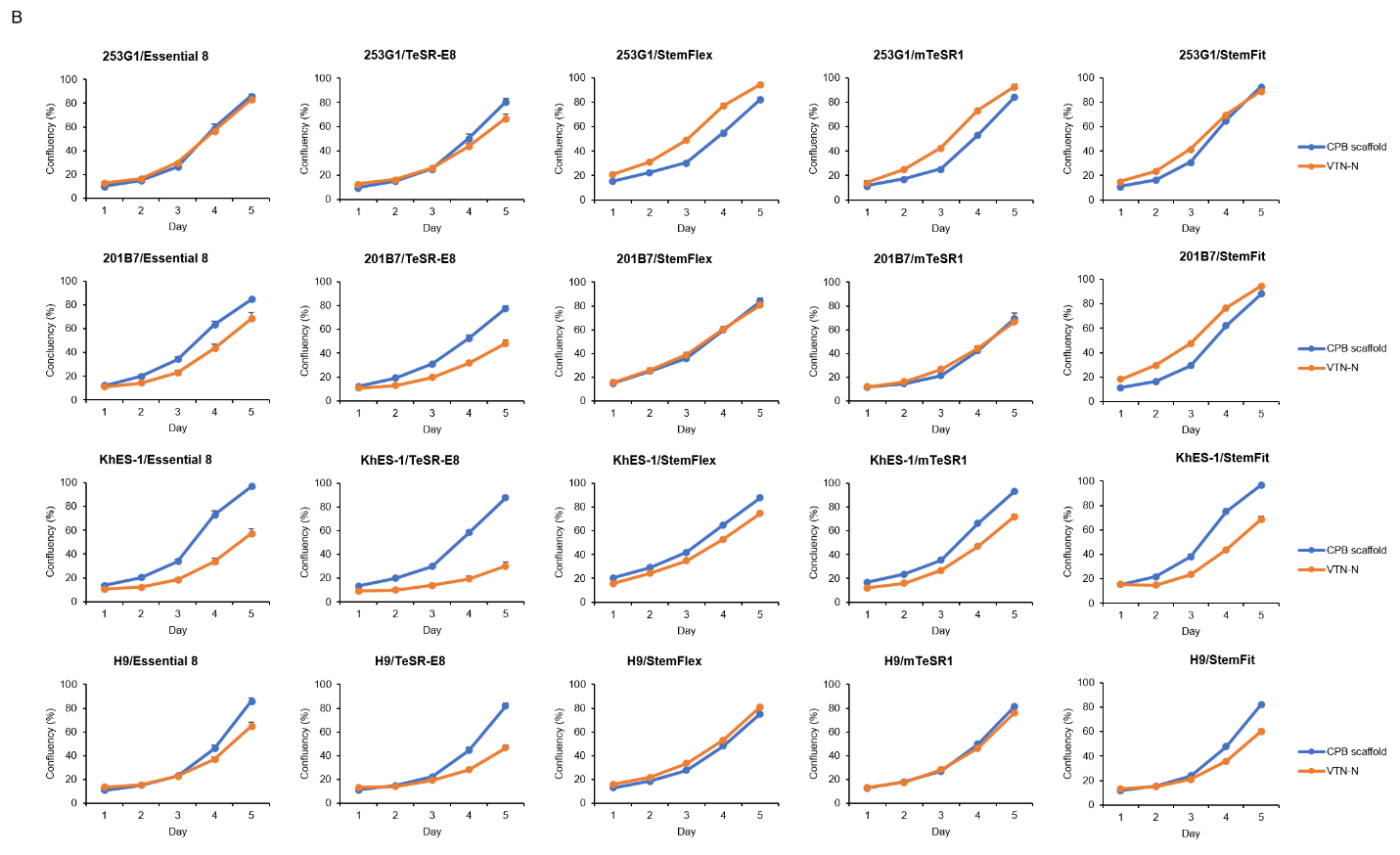
**

**Figure S4.** Examination of multiple media on CPB scaffold and VTN-N. Related to **Figure 5**. (A) Colony formation of 201B7 and H9 cells after **5** days in Essential 8, TeSR-E8, StemFlex, mTeSR1, or StemFit cultures. CPB scaffold or VTN-N was used as the culture substrate. All scale bars are 200 µm. (B) Cell proliferation curves during 5 days of culturing in Essential 8, TeSR-E8, StemFlex, mTeSR1, or StemFit after seeding 5 x 10^4^ 253G1 cells, 201B7 cells, KhES-1 cells or H9 cells per well on CPB scaffold or VTN-N coated 12-well plate. Data are presented as mean ± SD of three independent experiments.

**
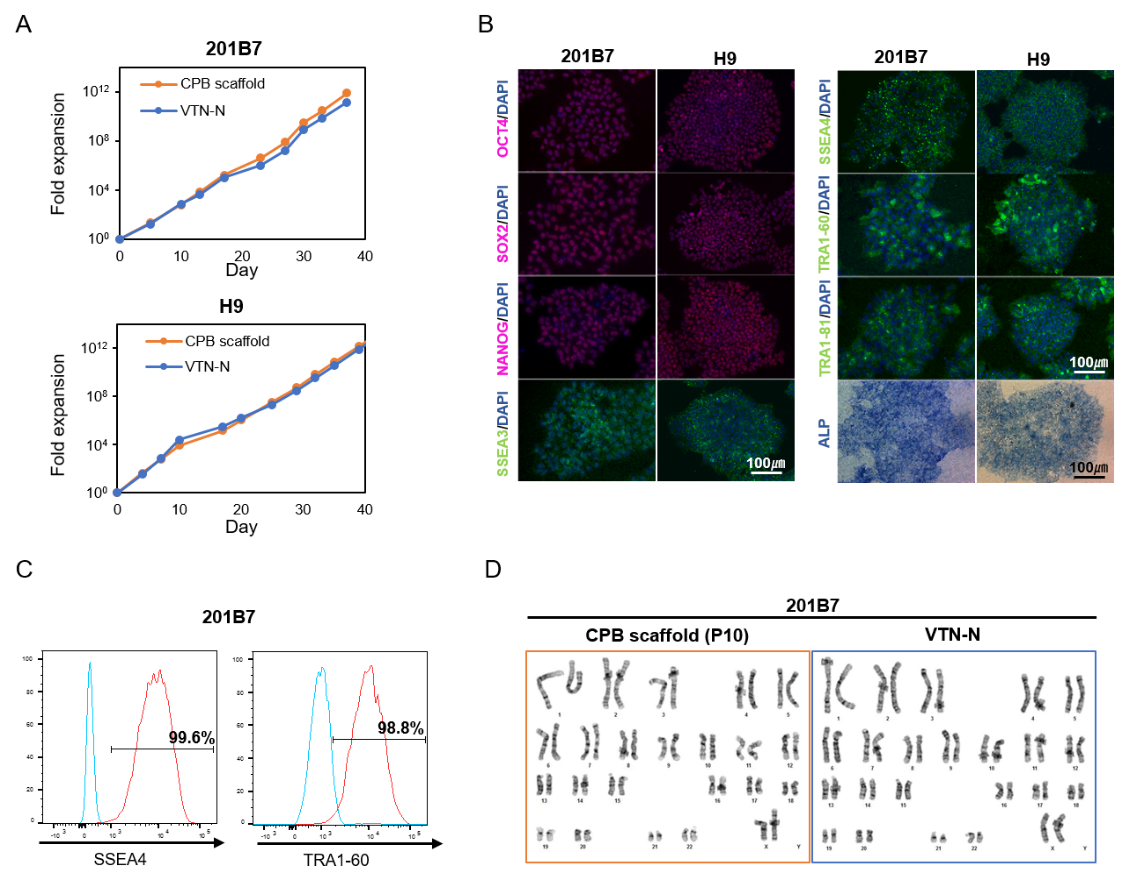
**

**Figure S5.** Characterization of hiPSCs and hESCs after long-term culture on CPB scaffold. Related to **Figure 6**. (A) Comparison of fold expansion rates of the 201B7 and H9 cell lines on CPB scaffold or VTN-N in Essential 8 medium. Fold expansion is shown in a one-logarithmic graph, and cell passages were performed more than 10 times. The orange circle on the graph shows the number of cells of 201B7 or H9 cultured on CPB scaffold, and the blue circle shows the number of cells cultured on VTN-N. (B) Pluripotency marker immunostaining and alkaline phosphatase (ALP) expression analysis. Pluripotency markers are red or green. Nuclear staining is performed with DAPI and shown in blue. ALP is blue in bright field. The scale bar represents 100 µm. (C) FACS analysis of pluripotency marker-positive 201B7 cells. Red and blue histograms show the stained and unstained control populations, respectively. At least 10,000 cells were measured for each sample. The vertical axis shows the mode standardized to 100. The percentage of maker-positive cells is indicated on each graph. (D) G-band karyotyping of the 201B7 cells maintained on CPB scaffold or VTN-N. Using 201B7 cells passaged 10 times or more on CPB scaffold or VTN-N, we observed normal chromosome numbers in 50 cells and performed karyotyping of 20 of them. A typical chromosomal image is shown.

**
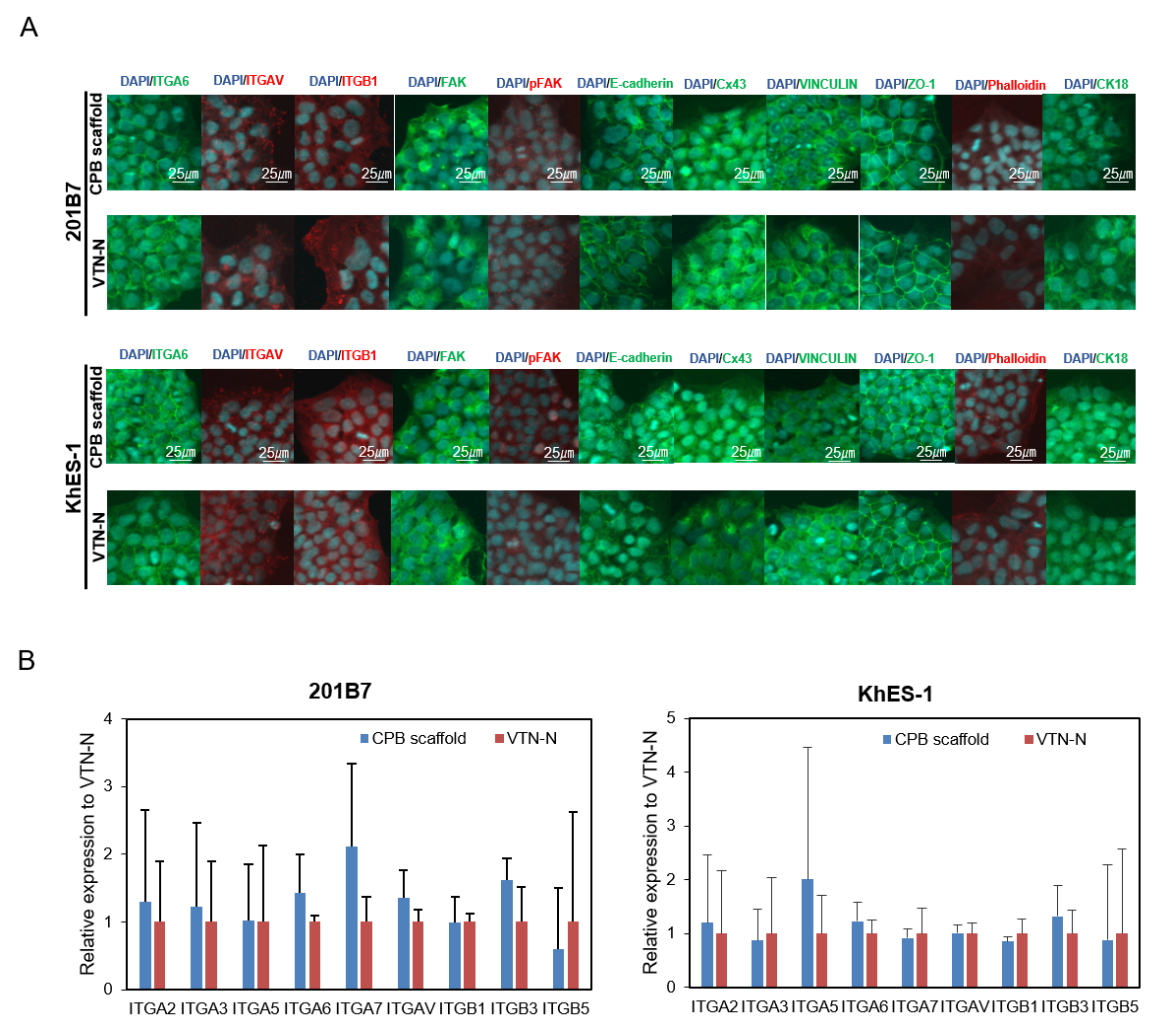
**

**Figure S6.** Immunostaining and RT-qPCR of integrins and other factors. Related to **Figure 7**. (A) Immunostaining of integrins, focal adhesion molecule, and cytoskeleton proteins of 201B7 cells and KhES-1 cells more than 10 passages on CPB scaffold or VTN-N. Integrins and other factors are shown in green, red, or orange, and DAPI is shown in blue. Scale bars indicated in the figure. (B) RT-qPCR results of the relative gene expression of Integrins normalized to expression on VTN-N. The vertical axis shows the fold change to VTN-N. Data are presented as mean ± SD of three independent experiments (**P*<0.05 compared to VTN-N).

**
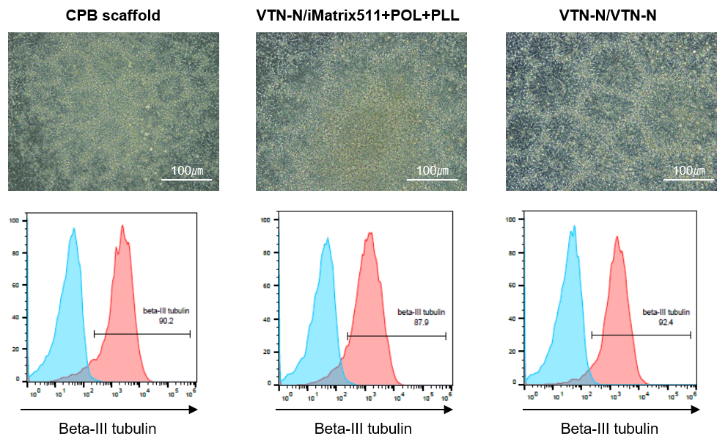
**

**Figure S7.** Differentiation of hiPSCs into neural progenitor cells on CPB scaffold. Representative images of cell/colony morphology and FACS population analysis histograms of beta-III tubulin-positive 253G1 cells on CPB scaffold (left), iMatrix511+POL+PLL (center) and VTN-N (right) at 12 day neunal differentiation related to **Figure 8**. The scale bar represents 100 µm. Red and blue histograms show the stained and unstained control populations, respectively. At least 10,000 cells were measured for the sample. The vertical axis shows the mode standardized to 100. The percentage of beta-3 tubulin-positive cells is indicated on the histogram.

**
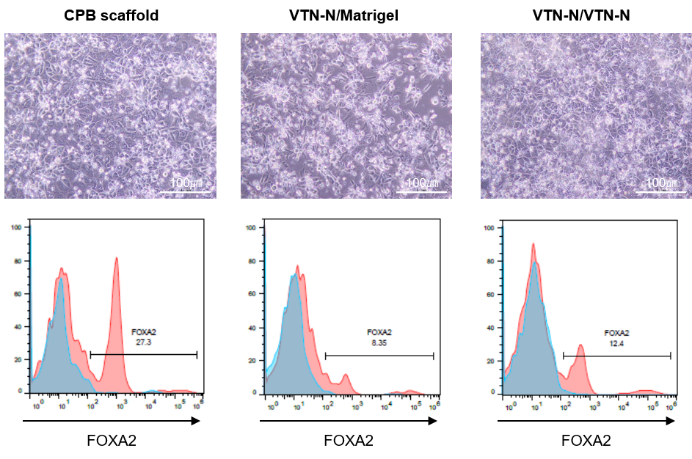
**

**Figure S8.** Differentiation of hiPSCs into hepatoblasts on CPB scaffold. Representative images of cell morphology and FACS population analysis histograms of FOXA2-positive 253G1 cells on CPB scaffold (left), Matrigel (center) and VTN-N (right) at 10 day differentiation related to **Figure 8**. The scale bar represents 100 µm. Red and blue histograms show the stained and unstained control populations, respectively. At least 10,000 cells were measured for the sample. The vertical axis shows the mode standardized to 100. The percentage of FOXA2-positive cells is indicated on the histogram.

**
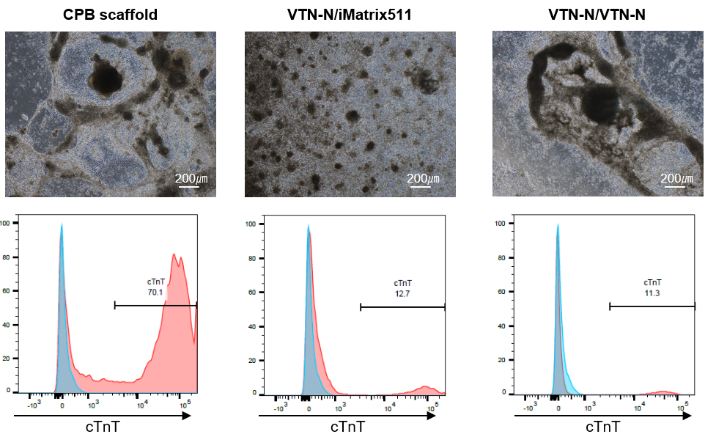
**

**Figure S9.** Differentiation of hiPSCs into cardiomyocytes on CPB scaffold. Representative images of cell morphology and FACS population analysis histograms of cTnT-positive 253G1 cells on CPB scaffold (left), iMatrix511 (center) and VTN-N (right) at 14 day cardiac differentiation related to **Figure 8**. The scale bar represents 200 µm. Red and blue histograms show the stained and unstained control populations, respectively. At least 10,000 cells were measured for the sample. The vertical axis shows the mode standardized to 100. The percentage of cTnT-positive cells is indicated on the histogram.

**Movie S1.** Beating 253G1-cardiomyocytes differentiated on CPB scaffold. Related to **Figure 8** **and S9**. This video shows beating of 253G1-cardiomyocytes differentiated on CPB scaffold at day 13.

**Table S1.** Properties of polyvinyl butyral-based substrate and other synthetic chemical substrates. Related to **Figure 2 and S1**. Contact angle of Tissue-Culture-polystyrene (TCPS), PVA, PEGMA, PNIPAM and PVB scaffold substrate.

| Synthetic polymer | Contact angle(°) |
| --- | --- |
| TCPS | 66 |
| PVA | 35 |
| PEGMA | 16 |
| PNIPAM | 60 |
| PVB | 84 |

**Table S2. Sequences of primers used in RT-qPCR of Integrins. Related to Figure 7 and S6.**

| Genes | Forward | Reverse | Size |
| --- | --- | --- | --- |
| *ITGA2* | GCAACTGGTTACTGGTTGGTT | GAGGCTCATGTTGGTTTTCATCT | 167 |
| *ITGA3* | CAAGGATGACTGTGAGCGGATG | CTGGTCTTCTGACCCTGACCA | 169 |
| *ITGA5* | GTCGGGGGCTTCAACTTAGAC | CCTGGCTGGCTGGTATTAGC | 152 |
| *ITGA6* | ATGCACGCGGATCGAGTTT | TTCCTGCTTCGTATTAACATGCT | 160 |
| *ITGA7* | CCAGTGTCCTCTGCTGAGAAG | GGACTTCACAGCTGAGTACTC | 174 |
| *ITGAV* | CATCTGTGAGGTCGAAACAGG | TGGAGCATACTCAACAGTCTTTG | 137 |
| *ITGB1* | GGAAATGGGACATTTGAGTGTGG | CTCTTCCTACAAACACACTGTCC | 185 |
| *ITGB3* | GGGGTAGGTTGGGAGAATGT | TCTGGGACAAAGGCTAAGGA | 156 |
| *ITGB5* | ACCAAGAGAGATTGCGTCGAGT | CAGCCTCCTGGTCATCTTTCA | 130 |

**Table S3. Sequences of primers used in RT-qPCR of differentiation markers of cardiac differentiation. Related to Figure 8D.**

| Genes | Forward | Reverse |
| --- | --- | --- |
| *hNKX2.5* | GTTGTCCGCCTCTGTCTTCT | TCTATCCACGTGCCTACAGC |
| *hTNNT2* | CAGAGCGGAAAAGTGGGAAGA | TCGTTGATCCTGTTTCGGAGA |
| *hCyclophilin A* | GTCCAGCATTTGCCATGGAC | GACAAGGTCCCAAAGACAGC |
